# Redefining Molecular Chaperones as Chaotropes

**DOI:** 10.3389/fmolb.2021.683132

**Published:** 2021-06-14

**Authors:** Jakub Macošek, Guillaume Mas, Sebastian Hiller

**Affiliations:** Biozentrum, University of Basel, Basel, Switzerland

**Keywords:** protein homeostasis, chaperone, biophysical mechanisms, chaotropicity, chaperone-client complexes, protein folding

## Abstract

Molecular chaperones are the key instruments of bacterial protein homeostasis. Chaperones not only facilitate folding of client proteins, but also transport them, prevent their aggregation, dissolve aggregates and resolve misfolded states. Despite this seemingly large variety, single chaperones can perform several of these functions even on multiple different clients, thus suggesting a single biophysical mechanism underlying. Numerous recently elucidated structures of bacterial chaperone–client complexes show that dynamic interactions between chaperones and their client proteins stabilize conformationally flexible non-native client states, which results in client protein denaturation. Based on these findings, we propose chaotropicity as a suitable biophysical concept to rationalize the generic activity of chaperones. We discuss the consequences of applying this concept in the context of ATP-dependent and -independent chaperones and their functional regulation.

## Introduction

Most proteins need to fold into a three-dimensional structure to perform their function, as encoded in their amino acid sequence (Anfinsen et al., 1961; Haber and Anfinsen, 1962; Anfinsen, 1973). While small proteins can fold efficiently, the vast majority of nascent protein chains needs to navigate a rugged potential energy surface, driven by the hydrophobic collapse and constrained by the crowded environment of the cell (Levinthal, 1968; Bryngelson and Wolynes, 1987; Wolynes et al., 1995; Onuchic and Wolynes, 2004; Bartlett and Radford, 2009). Thus, proteins can easily become trapped in local folding minima, from where they need to overcome free energy barriers to reach the correct native conformation. Folding *via* such intermediate states is considered to be the rule for proteins larger than 100 amino acids (Brockwell and Radford, 2007). In addition, even proteins that are capable of spontaneously reaching their native conformation may unfold under stress conditions. Folding intermediates or unfolded proteins are dysfunctional, prone to aggregation and may lead to fatal conditions that are a threat to the health of the cell (Knowles et al., 2014; Tittelmeier et al., 2020).

To tackle this challenge, protein homeostasis networks have evolved in all kingdoms of life (Hipp et al., 2019). They comprise of different molecular chaperones, as well as the ubiquitin-proteasome system (UPS) and the autophagy system. While UPS and the autophagy system play their functional role in degradation of expired proteins, chaperones are the key instrument of protein homeostasis. Chaperones not only facilitate folding of proteins, but also transport them, prevent their aggregation, dissolve aggregates or unfold misfolded proteins (Pelham, 1986; Ellis, 1987; Hemmingsen et al., 1988; Goloubinoff et al., 1989; Walter and Buchner, 2002; Hartl and Hayer-Hartl, 2009; Balchin et al., 2016; Goloubinoff, 2016; Wentink et al., 2019; Balchin et al., 2020; Burmann et al., 2020). Interestingly, a single chaperone can often perform several of these functions. For example, heat shock protein 70 (Hsp70, or DnaK in bacteria) participates in *de novo* protein folding, assembly of protein complexes and translocation across membranes to protein refolding, disaggregation, and degradation (Mayer and Gierasch, 2019). The underlying mechanism allowing a single chaperone to perform functions with such drastically different outcomes remains unclear.

Here, we develop a hypothesis addressing this question. We start out by summarizing the main cellular functions of chaperones and connecting them to protein folding theory. Then, we recapitulate recent structures of chaperone–client complexes, with a focus on bacterial systems. These connect the functional understanding of chaperone activity with structural insights and identify common patterns in the client dynamics. Finally, we extrapolate from these patterns to propose chaotropicity as a concept to describe the single biophysical activity underlying the diverse cellular functions of chaperones common to many or all chaperones.

## Cellular Functions of Chaperones and Their Connection to Protein Folding Theory

The traditional nomenclature to describe chaperone functions is based on their effective functionality in the cellular context. Depending on this context, a chaperone thus can act as a holdase, foldase, translocase, disaggregase, or unfoldase.

Holdase chaperones are typically ATP-independent chaperones, that merely associate with non-native client proteins for extended time periods to stabilize them and prevent their aggregation (Hall, 2020). Despite the fact that holdases do not directly fold proteins, their activity is indispensable as they protect vulnerable non-native states from aggregation. Studies revealing a broad clientome of holdases have illustrated their importance in protein folding (Haslbeck et al., 2004; Jarchow et al., 2008). Traditional representatives are the small heat shock protein (sHsp) family (Haslbeck et al., 2019), as well as a number of bacterial chaperones including cytosolic trigger factor (TF) and SecB, as well as periplasmatic Spy, Skp, and SurA (Bechtluft et al., 2010; Hoffmann et al., 2010; Goemans et al., 2014; Mas et al., 2019). Some holdases, such as TF, associate with ribosomes, thus comprising the first of the two chaperone layers participating in *de novo* protein folding (Frydman, 2001; Deuerling and Bukau, 2004; Kramer et al., 2004b; Kaiser et al., 2006; Merz et al., 2008). Holdases then transfer the nascent protein for active folding to the second layer of chaperones.

Active structural remodeling during *de novo* protein folding is the domain of ATP-dependent foldases. In bacteria, these are mainly the DnaK system and the GroEL/ES system (Hayer-Hartl et al., 2016; Rosenzweig et al., 2019). Both systems function similarly by cycling between ADP-bound and ATP-bound states that differ in affinity for non-native proteins. If the association rate of the binding to chaperone is greater than the aggregation rate and lower than the folding rate, the chaperones facilitate folding by kinetic partitioning (Diamond and Randall, 1997; Fedorov and Baldwin, 1997; De Los Rios and Barducci, 2014). Notably, DnaK and GroEL/ES systems differ in how they function mechanistically. While in the case of DnaK the folding occurs upon release, the group I chaperonin system GroEL/ES unfolds the client protein by expansion and then traps it in a cage, where the client protein collapses to fold (Hemmingsen et al., 1988; Lin and Rye, 2004; Lin et al., 2008; Sharma et al., 2008).

The third group of chaperones are translocases, which shuttle nascent proteins across membranes. Translocases are especially important in bacteria, where about a third of all proteins is exported from the cytoplasm and therefore needs to be translocated across the inner membrane. The main transport route for these proteins is the SEC pathway, using the key motor-protein SecA (Vrontou and Economou, 2004; Tsirigotaki et al., 2017). The molecular machine SecA converts chemical energy into mechanical force to translocate the unfolded nascent protein through the SecYEG membrane channel while maintaining the proteins unfolded. Nascent proteins may find SecA independently or be targeted to it by SecB and TF, but SecA associates with ribosome and interacts with nascent proteins directly as well (Huber et al., 2011; Wang et al., 2017).

The unfoldase function of chaperones is necessary to overcome free energy barriers in the case of nascent proteins trapped in local minima of their folding landscape or for the turnover of irreversibly misfolded proteins. Indeed, GroEL/ES was shown to begin its functional cycle with unfolding the client protein by expansion (Lin and Rye, 2004; Priya et al., 2013b; Mattoo and Goloubinoff, 2014), and similar unfolding by expansion was also described for DnaK (Sharma et al., 2010; Imamoglu et al., 2020). Moreover, a recent study of DnaK-assisted refolding of firefly luciferase suggests that initial unfolding is critical even for efficient folding of multi-domain proteins (Imamoglu et al., 2020). Overall, most chaperones have the capacity to destabilize protein structure (Sharma et al., 2009; Finka et al., 2016; Hiller, 2020).

If all the aforementioned activities of chaperones fall short to prevent proteins from aggregation, some chaperones still exhibit disaggregase activity, which allows them to untangle aggregates and refold the protein or target it for degradation (Sousa, 2014). Two major bacterial chaperone systems, Clp and DnaK, are capable of actively unraveling protein aggregates that would otherwise be aggregated irreversibly, and refold the proteins into their native conformation (Glover and Lindquist, 1998; Goloubinoff et al., 1999).

Two important observations support the notion that a single activity might underlie this large variety of chaperone functions in the cellular context. Firstly, for many chaperones with little client specificity – so-called general chaperones (Bose and Chakrabarti, 2017) - the major variable changing between particular cellular functions is modulation of client specificity by a co-chaperone or by subcellular localization. This implies that these general chaperones may use a single activity to perform their different cellular functions.

Secondly, functional studies indicate that different chaperones including DnaK, GroEL/ES, and Hsp90, unfold their client by expanding prior to facilitating their folding (Shtilerman et al., 1999; Ben-Zvi et al., 2004; Lin et al., 2008; Sharma et al., 2010; Walerych et al., 2010; Priya et al., 2013b; Mas et al., 2018), and some degree of unfolding is now emerging as the core aspect of the activity of many chaperones (Priya et al., 2013a; Finka et al., 2016; Jo et al., 2019). Such a chaperone activity is applicable to any of the cellular functions as unfolding the client gives it a chance to undergo renewed hydrophobic collapse.

**FIGURE 1:**
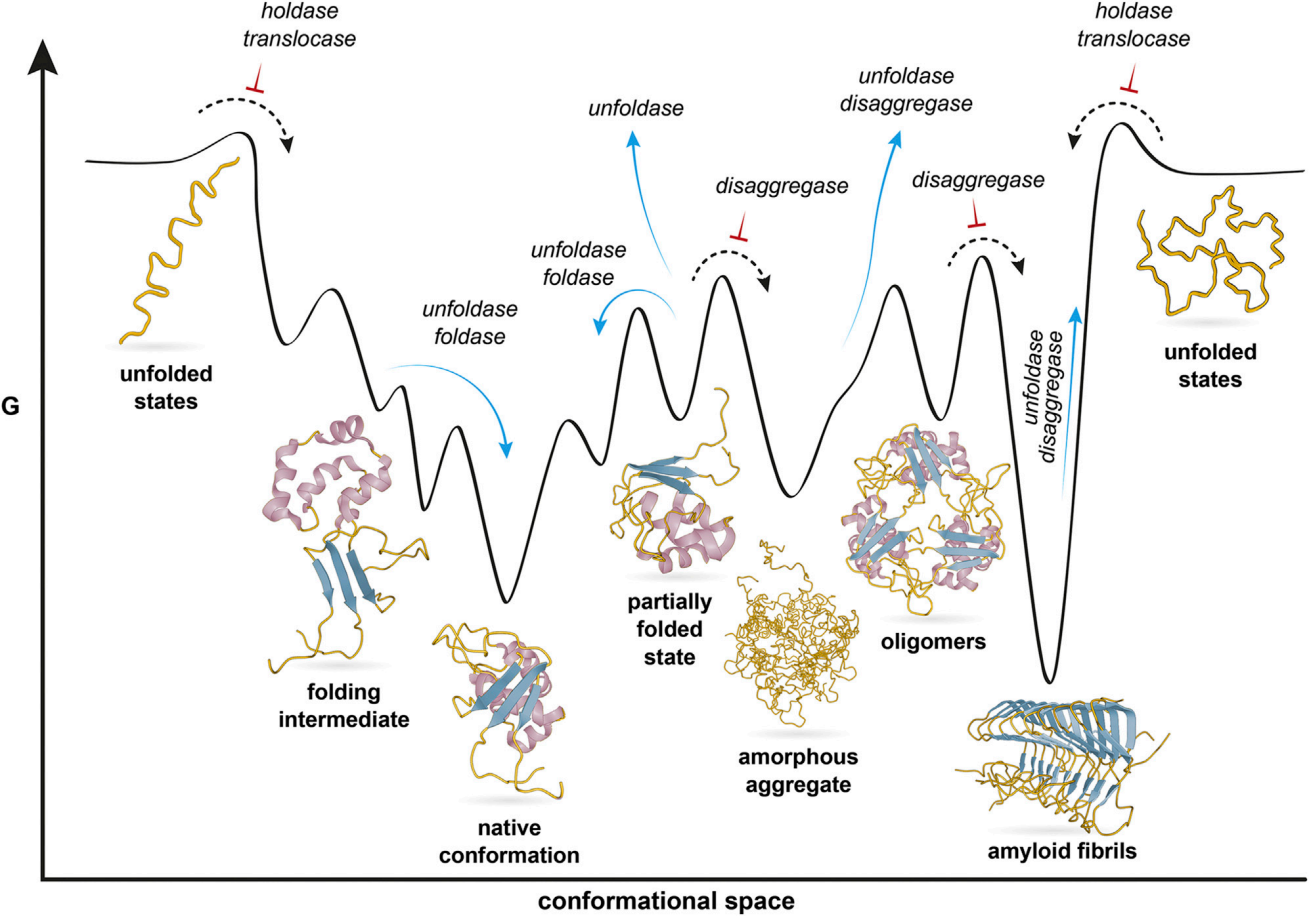
Cellular functions of chaperones in the context of a protein folding landscape. The protein polypeptide chain is shown yellow, with different secondary structure elements highlighted in purple and blue. The protein navigates a rugged free energy surface. The native conformation is one out of several local minima, representing different conformational states that are shown below the surface. Chaperones participate in a broad range of cellular processes which define a range of functions listed in italics. These navigate the protein along the energy landscape. Blue arrows indicate transitions that chaperones facilitate, whereas black arrows with red inversed T indicate transitions chaperones prevent. Figure modified from Jahn and Radford (2005).

The cellular functions of chaperones can thus be recast from the perspective of protein folding theory. Protein folding has been formulated as the problem of a nascent protein chain navigating in its conformational space on a funnel-shaped, rugged potential energy surface, with the eventual goal to attain its native conformation, a local minimum (Dill and MacCallum, 2012). Thereby, the ruggedness offers several local minima, which correspond to alternative structural states (Figure 1). From the perspective of protein folding, chaperones function in cellular folding processes by regulating transitions of the client protein between structural states on the potential energy surface. Since all cellular chaperone activities can be connected on a single folding landscape, chaperones in principle only need a single generic activity, that increases the free energy of the client. Each cellular chaperone function can then be viewed as the generic chaperone activity acting at specific positions of the potential energy surface to achieve the observed outcome.

## Lessons Learnt From Structures of Bacterial Chaperone-Client Complexes

**FIGURE 2:**
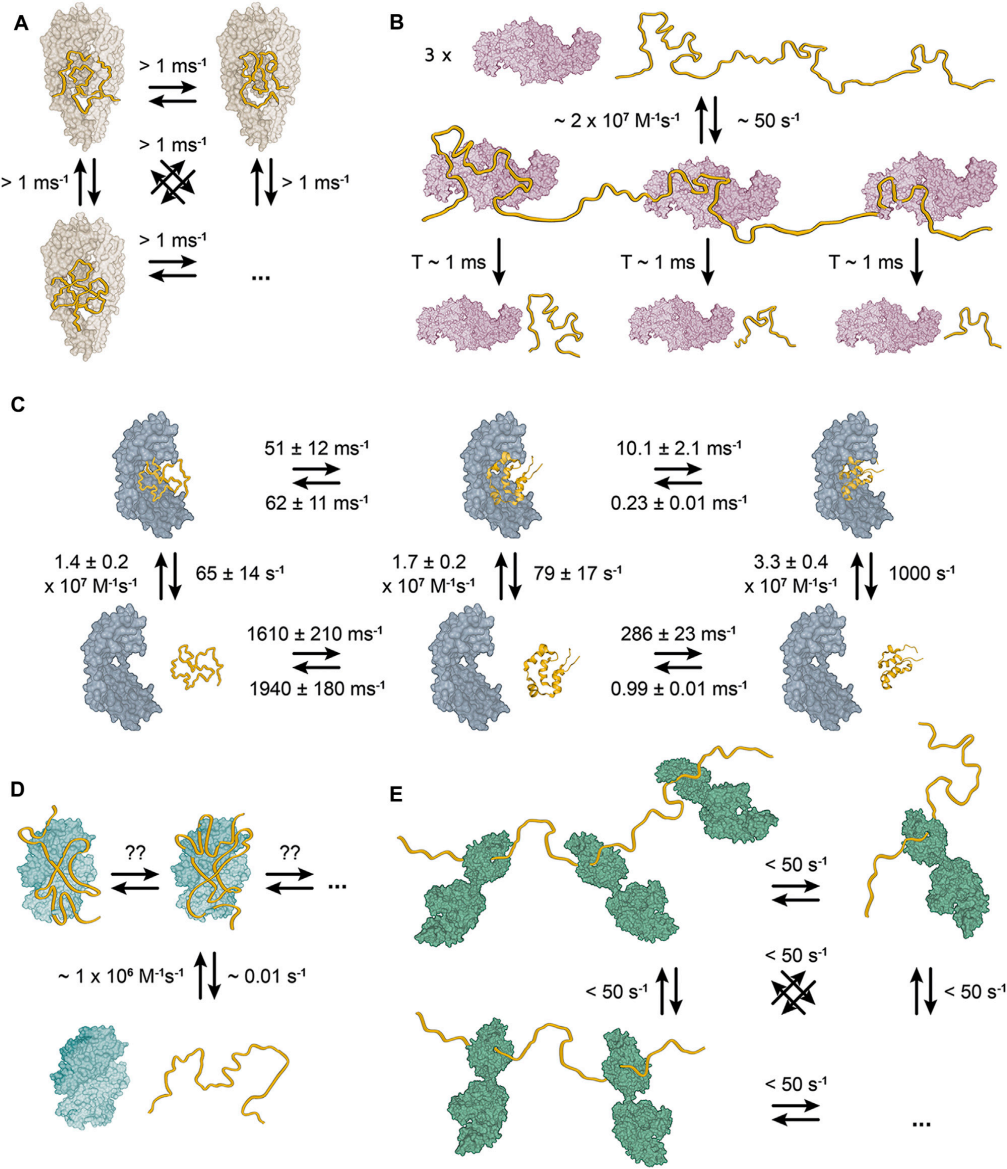
Structural models of bacterial chaperone-client complexes reveal dynamic interactions. **(A)** The Skp-OMP complex. The client binds as compact, flexible ensemble, which interconverts between individual conformations within 1 ms (Burmann et al., 2013). **(B)** The trigger factor–PhoA complex. A single molecule of PhoA interacts with three molecules of TF with short-lived interaction lifetimes of ∼1 ms (Saio et al., 2014). The kinetics reveal that the complex is also globally short-lived with a dissociation rate of ∼50 s^−1^. **(C)** The Spy-Im7 complex. The chaperone Spy binds its client Im7 as a dynamic ensemble of diverse conformations (He et al., 2016; Stull et al., 2016; Horowitz et al., 2018). The representative unfolded state (on the left), folding intermediate state (in the middle) and native state (on the right) interconvert with ms rates. However, the rates are slower for Spy-bound Im7 than for free Im7. **(D)** The SecB-MBP complex. One molecule of the client binds one SecB tetramer (Huang et al., 2016). No symmetry breaking of the SecB tetramer is observed upon binding of the full-length clients, which indicates that the resulting complex must be dynamic with the client rearranging on SecB surface on a very fast timescale. **(E)** The DnaK–hTRF1 complex. DnaK binds the client in an ensemble of globally unfolded conformations at various stoichiometric ratios (Lee et al., 2015; Sekhar et al., 2016; Rosenzweig et al., 2017).

In order to understand the cellular functions of chaperones mechanistically, it is crucial to employ biophysical descriptions of chaperone activity that regulate the transitions of proteins along the potential energy surface. The ideal starting point of such investigations are detailed structural descriptions of chaperone-client complexes as they provide direct snapshots of chaperones in action. Recent technological advances, particularly in solution NMR spectroscopy, have provided atomic resolution insights such complexes. In the following, we summarize such structural descriptions, including ATP-independent and ATP-dependent chaperones in order to reveal common features (Figure 2). We thereby focus on bacterial systems, because the most detailed descriptions are available for these, and because it can be assumed that the resulting conclusion can be generalized.

The earliest atomic-resolution characterization of a bacterial chaperone in complex with a full-length client protein was the study of bacterial chaperone Skp and the outer membrane proteins (OMPs) tOmpA and OmpX (Burmann et al., 2013). Skp is a holdase chaperone in one of two alternative pathways to transport OMPs in the periplasm to the outer membrane (Sklar et al., 2007). It is a 3 × 17 kDa trimer, that resembles a jellyfish (Kramer et al., 2004a; Walton and Sousa, 2004). Each monomer consists of a β-strand domain, which forms the body, and an extended coiled-coil domain, which forms the tentacles. The trimerization interface is located in the body of the trimer, whereas the α-helical tentacles define a central cavity that creates a protective environment for the client proteins (Burmann et al., 2013; Callon et al., 2014). The structural characterization of the Skp-OMP complexes revealed that the client binds as compact, but structurally disordered and highly conformationally flexible ensemble, in which the individual conformations interconvert within 1 ms (Figure 2A). Individual contacts between the client and the chaperone are weak and unspecific, but their avidity results in a high-affinity complex with a lifetime of more than 2 h. Upon binding to Skp, the clients remain in a highly conformationally flexible state, which allows them to sample more than 10^7^ conformations before their release from the chaperone (Burmann et al., 2013).

SurA is the principal chaperone in the second of the pathways to transport OMPs in the periplasm and its complexes with various OMPs have been recently structurally characterized (Marx et al., 2020). SurA is a ∼47 kDa chaperone consisting of three domains: the N and C-termini of the protein make up the core domain, while two peptidyl-prolyl isomerase domains (P1 and P2) comprise of the middle of segment of the protein chain (Bitto and McKay, 2002). The core domain and P1 form a platform connected to P2 by two flexible linkers, which allow the protein to alternate between multiple conformations (Calabrese et al., 2020). Characterization of the bound client-proteins revealed that similarly to Skp, SurA binds to the OMP in a disordered state (Calabrese et al., 2020; Marx et al., 2020).

The complex of TF and alkaline phosphatase (PhoA) is another complex that has been characterized at atomic resolution (Saio et al., 2014). PhoA is a ∼50 kDa periplasmatic enzyme, which can be unfolded and aggregation-prone in the cytosol (Valent et al., 1995). TF is a bacterial ribosome-associated holdase chaperone, that has a general function of protecting unfolded nascent proteins against aggregation (Hoffmann et al., 2010). It is a 48 kDa protein consisting of three domains adopting a dragon-like shape (Ferbitz et al., 2004). The N-terminal ribosome-binding domain (RBD) mediates ribosome interaction (Hesterkamp et al., 1997), the middle domain (PPD) has a peptidyl–prolyl isomerase activity (Hesterkamp and Bukau, 1996) and the C-terminal substrate-binding domain (SBD) carries the chaperone activity (Merz et al., 2006). TF exists in a fast monomer-dimer equilibrium, where the monomeric form is the active chaperone and the dynamic dimer is the storage form (Patzelt et al., 2002; Morgado et al., 2017). The interaction with the client PhoA causes a dissociation of the dimer, but full-length PhoA is too large for a single molecule of TF (Saio et al., 2014). On this basis, the structure of the TF–PhoA complex was determined as three individual TF bound to three fragments of PhoA (Saio et al., 2014). In the complexes, PhoA interacts predominantly with the SBD and to a lesser extent with the PPD. Each complex structure shows that a particular PhoA fragment binds in a unique conformation (Figure 2B). However, the same site of TF binds each of the PhoA fragments and NMR relaxation dispersion measurements show that the lifetime of a complex of TF and a single PhoA fragment is only ∼20 ms (Saio et al., 2014). Therefore, the TF-PhoA interaction is highly dynamic with the fragment of PhoA bound to a given molecule of TF constantly alternating.

A further milestone in our understanding of chaperone-client interactions came from the characterization of the complex of chaperone Spy and client protein colicin immunity protein 7 (Im7). Im7 is a bacterial immunity protein, which binds colicin E7 to inhibit its toxicity (James et al., 1996). Spy is a bacterial periplasmatic ATP-independent chaperone, which was identified in a screen for proteins that stabilize a mutant of Im7 (Quan et al., 2011). It is a ∼16 kDa protein that forms a cradle-shaped dimer (Kwon et al., 2010; Quan et al., 2011). Im7 binds the concave surface of Spy dimer, but determining a crystal structure of the complex did not reveal the structure of chaperone-bound Im7, because the electron density of Im7 was of insufficient quality (Horowitz et al., 2016). Subsequently, NMR spectroscopy confirmed the conformational flexibility of bound Im7 and revealed its interaction site on the concave surface of Spy (He et al., 2016). Additionally, the same binding mode was observed in the complex of Spy with the Fyn SH3 domain (He and Hiller, 2018). Spy supports the conformational flexibility of the bound client to such an extent that folding of Im7 while bound to Spy was reported (Stull et al., 2016), despite the fact that Spy is an ATP-independent chaperone that does not undergo any large conformational changes. The key aspect of Spy facilitated folding of Im7 is that the folding rate is significantly decelerated compared to the folding of free Im7 (Figure 2C).

The largest structurally well-characterized chaperone–client complex is the complex of the chaperone SecB with PhoA. SecB is a bacterial cytosolic ATP-independent holdase chaperone responsible for maintaining bacterial secretory proteins in unfolded state and delivering them to SecA in the SEC translocation pathway (Hartl et al., 1990). Additionally, SecB is also a general holdase chaperone like TF (Ullers et al., 2004). SecB exists as dimer of a dimers, where each monomer is a single-domain α/β fold protein with a molecular weight of 17.5 kDa (Hartl et al., 1990; Xu et al., 2000; Dekker et al., 2003). Dimer of dimers means that in each dimer one monomer is always equivalent to a monomer in the other dimer. In the NMR spectra of SecB, this property results in doubling of the peaks for each amino acid of the monomer (Huang et al., 2016). In contrast to TF, client binding does not induce dissociation of the SecB tetramer and so, a single molecule of PhoA binds one tetramer of SecB. In the complex, PhoA wraps around the SecB tetramer in an elongated conformation. Like in complex with TF, each PhoA binding site interacts with SecB in a unique conformation. However, different fragments of PhoA may interact with the same site of SecB and the authors do not note any further symmetry breaking in the complex of SecB with full length PhoA as well as they state that in the complex each PhoA site can bind any SecB site (Huang et al., 2016). This means that the SecB-PhoA interaction is highly dynamic with individual PhoA sites constantly alternating between the same binding sites on SecB, like PhoA sites between different molecules of TF in the TF-PhoA complex. Besides the PhoA-SecB complex the authors also characterized MBP-SecB complex, which revealed the same binding mode (Figure 2D).

DnaK (Hsp70 in eukaryotes) is one of the key general foldase chaperones in all kingdoms of life (Rosenzweig et al., 2019). To function in a large variety of cellular processes, DnaK associates with numerous nucleotide exchange factors (NEFs) and diverse co-chaperones from the Hsp40 protein family known as J domain proteins (JDPs). DnaK consists of two domains – the nucleotide binding domain (NBD), which harbors its ATPase activity (Flaherty et al., 1990), and the SBD, which consists of a β-sheet sandwich (SBDβ) and an α-helical lid (SBDα) (Zhu et al., 1996). Nucleotide binding controls the allosteric cycle of DnaK, which alternates between an open and a closed conformation of the SBD. In the ATP-bound state, SBDα is dissociated from SBDβ and both are docked on the NBD, resulting in lower affinity of SBD toward clients (Takeda and McKay, 1996; Swain et al., 2007; Zhuravleva and Gierasch, 2011; Kityk et al., 2012; Qi et al., 2013). Upon ATP hydrolysis, DnaK transitions to the ADP-bound state in which SBDα encloses the client in the cleft of SBDβ and has high affinity toward clients (McCarty et al., 1995; Zhuravleva et al., 2012). In this state SBD and NBD do not interact and tumble as independently as their connecting linker allows (Bertelsen et al., 2009). The first chaperone-client complex of DnaK, which was characterized structurally in detail was the complex of DnaK with the SH3 domain of drkN (Lee et al., 2015). Although the characterization did not result in a structural model of the chaperone–client complex, it provides the crucial observation that the SH3 domain interacts with DnaK in a dynamic ensemble of multiple globally unfolded states (Figure 2E). The interaction resembles the Skp-OMP complexes and Spy–Im7 complex, but SH3 alternates between the individual conformations on a timescale slower than for the Skp-OMP complexes (>>20 ms). Subsequently, the characterization of DnaK in complex with hTRF1 painted a similar picture (Sekhar et al., 2015; Sekhar et al., 2016; Rosenzweig et al., 2017). hTRF1 also binds DnaK in a dynamic ensemble of multiple globally unfolded states, that exchange on a slower timescale comparing to the Skp-OMP complexes. Additionally, the characterization of the DnaK-hTRF1 complex provides three more important insights. Firstly, hTRF1 binds DnaK as an ensemble regardless of the nucleotide state of DnaK, secondly, DnaK binds the client at 1:1, 1:2, and 1:3 client:DnaK stoichiometric ratios and thirdly, the DnaK residues involved in the interaction with hTRF1 are also conformationally flexible (Sekhar et al., 2015; Sekhar et al., 2016; Rosenzweig et al., 2017).

Taken together, these bacterial chaperone-client complexes characterized at atomic resolution make up a comprehensive dataset (Figure 2), which provides three key conclusions: (i) chaperone-client interactions are generally highly dynamic, with fast dissociation constants, both globally and locally, (ii) chaperone-bound clients are conformationally highly dynamic and populate interconverting conformational ensemble states on the chaperone surface. (iii) although chaperone-client interactions are widely believed to be mediated by hydrophobic contacts, this cannot be generalized from the complexes discussed above. While the complexes of SecB and TF appear dominated by hydrophobic interactions, Spy–Im7 and Skp–OMP feature both electrostatic contacts as well as hydrophobic interactions (Qu et al., 2007; Saio et al., 2014; Huang et al., 2016; Koldewey et al., 2016). The importance of both hydrophobic and electrostatic interactions in chaperone–client complexes has also been shown in a eukaryotic chaperone (Sučec et al., 2020).

The chaperone-client complexes thus demonstrate how chaperones destabilize the structure of clients by highly dynamic interactions, in line with data from functional studies (Finka et al., 2016). As seen on the examples of DnaK and Spy, the interaction is often selective for unfolded and non-native client states, which are thus stabilized relative to the native state. Notably, the binding of a chaperone to a client leads to an overall increase in stability for the resulting client – chaperone complex relative to the client alone, but this does not automatically indicate that the partially folded client is itself stabilized. Therefore, experiments that probe the result complexes tend to observe a stabilization (Mashaghi et al., 2016), while in experiments that monitor the clients selectively a destabilization is detected. In the case of Spy, which allows its client Im7 to fold while chaperone-bound, the folding rates of Im7 are significantly reduced as a result of the stabilization of the non-native states (Stull et al., 2016). Collectively, these structural and functional studies reveal as a putative general mechanism that chaperones thermodynamically destabilize protein structure by stabilizing non-native states.

## Toward a Unifying Biophysical Principle Underlying Chaperone Function

During the folding process, proteins need to sample conformationally highly flexible states in order to reach a distant minimum corresponding to the native conformation on the potential energy surface. These folding transition states are thermodynamically unfavorable, because they expose hydrophobic residues to the solvent which requires ordering of the surrounding solvent molecules. Upon folding according to the hydrophobic collapse model, hydrophobic residues gather in the protein core and reduce their exposure to the solvent. The entropy of the polypeptide chain decreases upon folding, which limits its opportunity to explore its conformational space, but the overall entropy of the system increases due to the release of the solvent making hydrophobic collapse thermodynamically favorable. Chaperones provide interaction surfaces that can thermodynamically stabilize proteins in highly flexible transition states, thus delaying the hydrophobic collapse and allowing the protein to explore its conformational space better. In the characterized chaperone-client complexes (Figure 2), the interaction with chaperones selectively stabilizes conformationally flexible non-native states of the client-protein, which in turn destabilizes the highly structured states, including the native conformation or aggregated states.

Importantly, such an effect of chaperones bears a striking resemblance to the well-characterized effect of chemical chaotropes (Hiller, 2020). Urea and other small co-solutes potently disrupt native structures of biomolecules (Hamaguchi and Geiduschek, 1962). Chaotropes counteract the hydrophobic collapse by directly or indirectly increasing the solubility of the hydrophobic residues, thus destabilizing protein native structure as well as protein aggregates. Proteins dissolved in chaotropes display large conformational flexibility with high conformational entropy (Ball and Hallsworth, 2015). This entropy increase counteracts the entropy decrease from the ordering of the solvent molecules caused by solvent exposure of the hydrophobic residues. The exact mechanism of chaotropic denaturation is likely a combination of direct interactions of the chaotrope with the hydrophobic regions and tight interactions of the chaotrope with water molecules in bulk solvent, reducing the amount of available water molecules for the solvation of the protein (Bennion and Daggett, 2003; Ball and Hallsworth, 2015).

Several indications in the experimental data of chaperone-client complexes suggest that chemical chaotropes and chaperones could indeed share similar mechanisms of action. Chaperones have been shown a source of entropy to their client protein in the Im7-Spy and Skp-OMP complexes, where binding of the client increased the client’s conformational flexibility (Burmann et al., 2013; He et al., 2016). The chaperone Spy supports the conformational flexibility of the bound client, allowing it to explore the conformational space sufficiently enough to fold while bound (Stull et al., 2016) and ITC measurement of the Im7–Spy interaction clearly show that the binding is driven by entropy increase (He et al., 2016). Furthermore, the client binding site of DnaK is conformationally flexible, suggesting that DnaK may also increase conformational flexibility of the client upon binding (Rosenzweig et al., 2017; Yang et al., 2017). The chaperones can thus increase the entropy of the client, shifting the client’s equilibrium away from the native state.

Besides these similarities, we also expect functional differences between small molecule chaotropes and chaperones. Small molecule chaotropes achieve chaotropicity by modulating the entire volume of a bulk solution due to their high molar concentrations. The same effect is inconceivable for chaperones as they function at orders of magnitude lower concentrations. Chaperones form pockets and grooves in which the solution may have drastically altered physicochemical properties in comparison to the bulk solution. It is thus conceivable to imagine a pocket with chaotropic properties. The formation of chaotropicity pockets at the chaperone surface thus allows them to create highly concentrated chaotropic environment even at stoichiometric concentrations. The residues comprising the inner surface of the pockets are pivotal to formation of chaotropic pockets and the chaotropicity of these pockets could be modulated by conformational changes. Mechanisms to regulate the activation of chaperones pockets have been shown for ATP-independent chaperones, such as regulation by different transition mechanisms such as oligomer disassembly, order-to-disorder transition or coupled folding/oligomerization (Reichmann et al., 2012; Haslbeck and Vierling, 2015; Suss and Reichmann, 2015; Mas et al., 2020). Such transitions drastically modulate the surface accessibility to the client proteins, providing a potential mechanism for the regulation of chaotropicity in ATP-independent chaperones.

**FIGURE 3:**
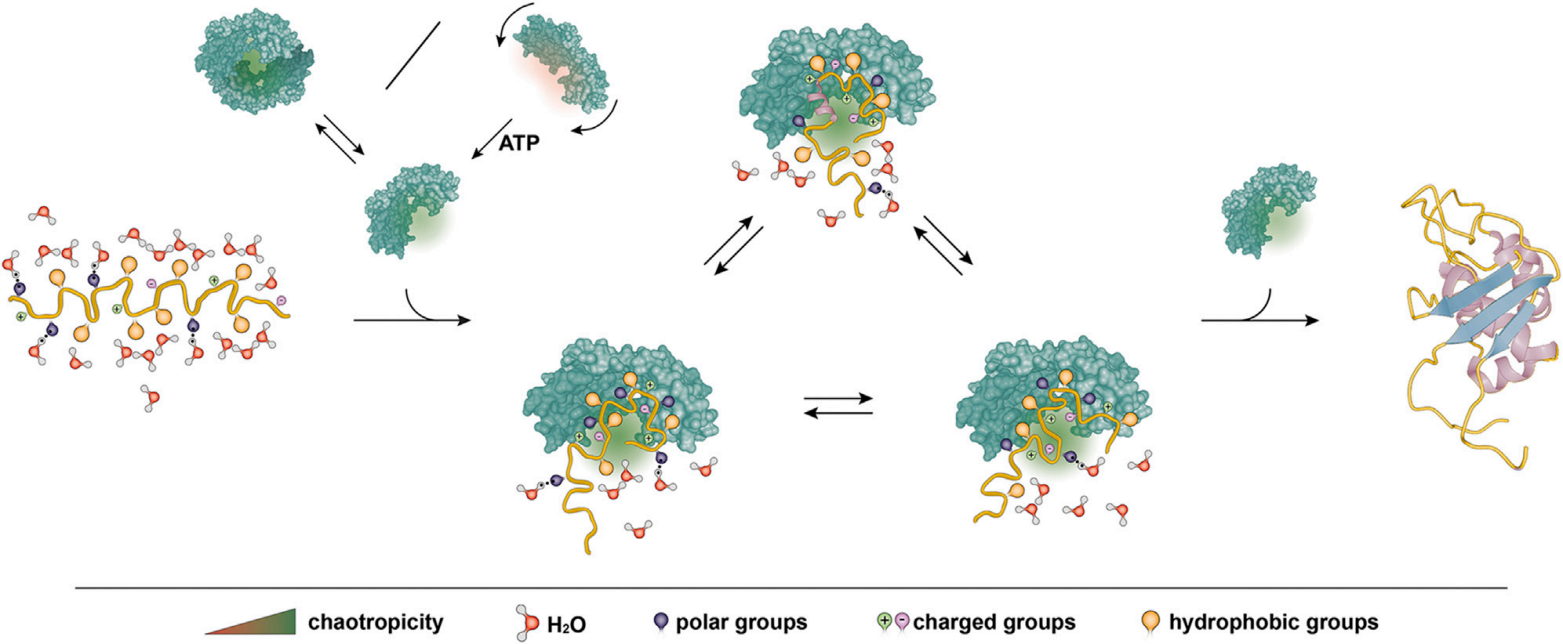
Proposed model of chaotropicity underlying generic activity of chaperones. A client protein backbone is shown in yellow with different side chain groups and solvating water molecules as indicated. A chaperone is shown in green. The chaperone features a chaotropic pocket to bind and stabilize the protein, protecting it from premature hydrophobic collapse into a misfolded state and allowing it to explore the conformational space. The protein may fold into its native structure on the chaperone surface or upon release. Chaotropicity stems from the amino acids in the pocket surface and may thus be regulated for example by altering the accessibility of the pocket through dimerization or by ATP-regulated conformational changes.

From these considerations, a direct step leads to chaperones that couple chaperone activity to ATP binding and hydrolysis. There are several conceivable mechanisms in which ATP hydrolysis could regulate chaotropicity of chaperones. Hsp90 is an ATP-dependent dimeric chaperone with a clamp-like structure and may provide the first example. ATP triggers large-scale conformational changes of Hsp90, which result in closing of the clamp, but interestingly that does not encapsulate the client protein, rather it creates a larger continuous bipartite binding surface (Ali et al., 2006; Street et al., 2011). Similarly, in GroEL ATP induces conformational changes that alter the client interaction surface, although not by dividing it, rather by changing the properties of the surface as a result of exchanging the residues exposed on the inner surface of the barrel due to the rotation of the chaperone monomers (Tang et al., 2006; Horwich et al., 2007; Villebeck et al., 2007; Tang et al., 2008; Horwich et al., 2009; Jewett and Shea, 2010). Thus, ATP-induced conformational changes may offer a way to directly regulate chaotropicity by perturbing the surface of the pocket where the client docks. Additionally, modular assembly of the pocket as outlined on the example of Hsp90 allows for residual chaperone activity in absence of ATP as each module would retain its chaotropicity (Figure 3). Such a residual activity in the absence of ATP was observed for many ATP-dependent chaperones (Wiech et al., 1992; Cho and Bae, 2007; Rao et al., 2010; Priya et al., 2013c; Mas et al., 2018).

In the cases of Hsp70 and Hsp90, the ATP cycle is regulated by numerous co-chaperones, which present a potential to further regulate chaperone chaotropicity. Co-chaperones are the key element in chaperone specificity (Bose and Chakrabarti, 2017), thus providing a possibility for function-dependent regulation of chaotropicity. Chaperones such as Hsp90 are commonly found in multiple organelles as well, which may mean different physicochemical properties of the surrounding solution that would inherently alter the chaotropicity of the chaperone. Indirect regulation of chaotropicity by co-chaperones could be a potent way to regulate chaperone chaotropicity to achieve the same effect in different environments.

In summary, chaperone chaotropicity provides a theoretical framework to explain previous experimental data as well as a thermodynamic description of generic chaperone activity for ATP-dependent and -independent chaperones. In contrast to existing models explaining the mechanism of chaperone functions, chaotropicity by default describes client proteins as multistate ensembles, which reflects more accurately the dynamic nature of chaperone–client complexes. The hypothesis presumes a similarity in the molecular mechanism of chaotropicity between small molecule chaotropes and chaperones and the extent of this similarity needs to be tested experimentally. Nevertheless, due to differences in complexity and effective range of concentration between chemical chaotropes and chaperones, the chaperone chaotropicity model also postulates that chaperones exert chaotropicity in a unique form of chaotropic pockets that can be tuned upon conformational change. Considering the crucial roles of chaperones for the health of any organism, a full rationale for their biophysical principles will advance our understanding of homeostasis as well as open new avenues for translational research.
